# Regulation of heme biosynthesis via the coproporphyrin dependent pathway in bacteria

**DOI:** 10.3389/fmicb.2024.1345389

**Published:** 2024-03-21

**Authors:** Hadia Aftab, Rebecca K. Donegan

**Affiliations:** Department of Chemistry, Barnard College, New York, NY, United States

**Keywords:** heme, heme biosynthesis, coproporphyrin dependent pathway, heme synthesis regulation, prokaryotic heme synthesis

## Abstract

Heme biosynthesis in the Gram-positive bacteria occurs mostly via a pathway that is distinct from that of eukaryotes and Gram-negative bacteria in the three terminal heme synthesis steps. In many of these bacteria heme is a necessary cofactor that fulfills roles in respiration, gas sensing, and detoxification of reactive oxygen species. These varying roles for heme, the requirement of iron and glutamate, as glutamyl tRNA, for synthesis, and the sharing of intermediates with the synthesis of other porphyrin derivatives necessitates the need for many points of regulation in response to nutrient availability and metabolic state. In this review we examine the regulation of heme biosynthesis in these bacteria via heme, iron, and oxygen species. We also discuss our perspective on emerging roles of protein-protein interactions and post-translational modifications in regulating heme biosynthesis.

## 1 Introduction

**FIGURE 1:**
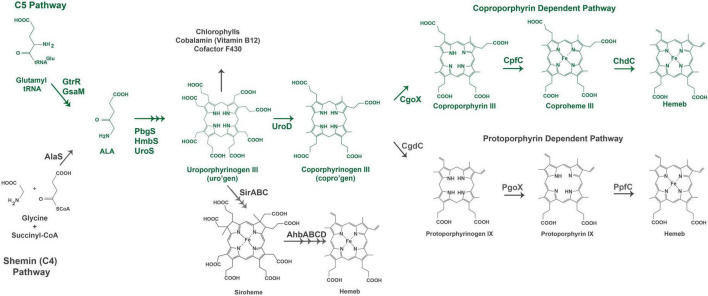
Heme biosynthesis pathways. The pathway used by Gram-positive bacteria is colored in green. The acronyms for heme biosynthesis genes are defined in Table 2.

Many of the bacteria of the Bacillota (formerly Firmicutes) and Actinomycetota (formerly Actinobacteria), herein referred to collectively under their previous designation of Gram-positive bacteria, utilize a heme biosynthesis pathway that differs in its terminal enzymes from the major pathway utilized by Gram-negative bacteria and eukaryotes (Dailey et al., 2017). This pathway progresses through a coproporphyrin intermediate and is termed the coproporphyrin dependent pathway (CPD). Of the monoderm Gram-positive bacteria, ∼70% use the CPD for heme biosynthesis (Dailey et al., 2015). In some monoderm bacteria, the terminal heme biosynthesis enzyme coproheme decarboxylase (ChdC) and other enzymes of the CPD are found, but the CPD does not appear to be the predominant heme biosynthesis pathway across these phyla. In the Chloroflexota, for example, combinations of enzymes of the protoporphyrin dependent pathway (PDP), Siroheme and CPD pathways are found, with some species having enzymes from all three pathways (Kim et al., 2021). Though several genomes have the terminal heme synthesis enzyme coproheme decarboxylase (ChdC see Figure 1), no species that have been found to have a complete heme biosynthesis pathway utilizes only the CPD pathway (Kim et al., 2021). The first enzyme unique to the CPD, coproporphyrinogen III oxidase (CgoX see Figure 1), and the last, ChdC, have also been found in multiple bacteria of the Deinococcota, suggesting that the CPD may be utilized by these bacteria, though further studies are needed to verify that heme is synthesized via this pathway (Dailey et al., 2015). In the diderm bacteria, the CPD is much less prevalent with only the Planctomycetota and Acidobacteria phyla reported as having ChdC in present the majority of the genomes analyzed by Dailey and colleagues (Dailey et al., 2015). Altogether, a majority of the monoderm Gram-positive bacteria of the Bacillota and Actinomycetota utilize the CPD, while most other monoderm bacteria and the diderm bacteria utilize other pathways such as the siroheme or PPD pathways.

Current knowledge of the regulation of heme biosynthesis in the Gram-positive bacteria reveals regulation at multiple points in the pathway and in response to various stimuli. This is to be expected as heme is an essential cofactor for many of these bacteria. Heme is a cofactor in proteins necessary for respiration, detoxification of reactive oxygen (ROS) and nitrogen species, and for gas sensing and transport (Choby and Skaar, 2016; Dailey et al., 2017; Donegan et al., 2019). The heme precursor uroporphyrinogen III is also a precursor for synthesis of cobalamin, which is synthesized *de novo* in some Gram-positive bacteria. Furthermore, heme synthesis in these bacteria requires glutamate, as glutamyl tRNA for porphyrin synthesis, and iron thus bridging acid and protein metabolism and iron homeostasis pathways (Dailey et al., 2017; Layer, 2021). The many uses of heme in the cell, the shared pathway with other porphyrin derivatives, and the various pathways linked via heme metabolism all offer potential points of regulation. Indeed, we see regulation in response to external stimuli including ROS, and in response to oxygen, iron, and heme levels. These responses are not universal and in some Gram-positive genera regulatory mechanisms have not yet been discovered, leaving the door open for new discoveries in heme synthesis regulation. Discussed within this review are the regulatory mechanisms of heme biosynthesis in Gram-positive bacteria via heme, iron, and oxygen levels. Additionally, we will discuss the emerging evidence for the role of protein-protein interactions and post-translational modifications (PTMs) in regulating heme biosynthesis.

### 1.1 Pathways of heme biosynthesis

Heme biosynthesis pathways are found in most organisms that can live aerobically and in organisms from all three domains of life. Heme in these organisms exists as many forms with heme*b* being the most abundant and a precursor for other forms of heme found in cytochromes. This includes the heme*a* and heme*o*, heme*d* which has a spiro-lactone modification, and the covalently attached heme*c*. Here we give a brief overview of the known heme biosynthesis pathways of heme*b* (referred to herein as heme) as these pathways have been previously reviewed in detail (Choby and Skaar, 2016; Dailey et al., 2017; Layer, 2021; Dailey and Medlock, 2022). To date, three distinct pathways for the *de novo* biosynthesis of heme have been discovered (Choby and Skaar, 2016; Dailey et al., 2017). These pathways are distinguished by the synthesis of heme from the uroporphyrinogen III intermediate (Figure 1). In the most ancient pathway, siroheme is synthesized from uroporphyrinogen III and then converted to heme. This siroheme dependent pathway is found in archaea and in some sulfate reducing bacteria. The coproporphyrin dependent pathway (CPD) likely evolved after the siroheme pathway and was discovered only recently by Dailey et al. (2010) and Dailey et al. (2015) to be the pathway utilized almost exclusively by the Gram-positive bacteria. The protoporphyrin dependent pathway (PPD) also evolved after the siroheme dependent pathway, but was for a time the only known heme biosynthesis pathway and believed to be universal (Dailey et al., 2017; Zámocký et al., 2023). The PPD is probably the most well studied as it is shared by eukaryotes and Gram-negative bacteria (Dailey et al., 2017). The synthesis of the porphyrin precursor 5-aminolevulinic acid (ALA) also occurs via two distinct pathways, either by the Shemin (or C4) pathway or via the C5 pathway. The synthesis of uroporphyrinogen III from ALA is shared by heme biosynthetic organisms, and bacteria and eukaryotes that use the CPD or PPD share the pathway from ALA to coproporphyrinogen III (Figure 1). The ALA biosynthesis and heme biosynthesis pathways most used by different organisms are summarized in Table 1 and the abbreviations for the proteins in Figure 1 are defined in Table 2.

**TABLE 1:** Summary of ALA and heme biosynthesis pathways commonly found in different organisms.

Organism	ALA pathway	Heme pathway
Archaea	C5	Siroheme
Gram-positive bacteria	C5	CPD
Gram-negative bacteria	C5	PPD
Alphaproteobacteria	Shemin	PPD
Fungi	Shemin	PPD
Plants	C5	PPD
Metazoans	Shemin	PPD

C5, C5 ALA biosynthesis pathway that utilizes glutamyl-tRNA; CPD, coproporphyrin dependent pathway; PPD, protoporphyrin dependent pathway.

**TABLE 2:** Heme biosynthesis pathway enzymes and acronyms used in Figure 1 and Tables 3, 4.

Enzyme	Abbreviation	Pathway	Other abbreviations
**ALA synthesis**			
Glutamyl-tRNA reductase	GtrR	C5	HemA/GtrA
Glutamate semialdehyde aminomutase	GsaM	C5	HemL/GSAM
ALA synthase	AlaS	Shemin	HemA
**Uroporphyrinogen synthesis**			
Porphobilinogen synthase	PbgS	Core	HemB/ALAD/PBGS
Hydroxymethylbilane synthase	HmbS	Core	HemC/HMBS/PBGD
Uroporphyrinogen synthase	UroS	Core	HemD/UROS
**Heme synthesis from uroporphyrinogen**			
Uroporphyrinogen decarboxylase	UroD	CPD/PPD	HemE/UROD
Coproporphyrinogen oxidase	CgoX	CPD	HemY
Coproporphyrin ferrochelatase	CpfC	CPD	HemH/HemZ
Coproheme decarboxylase	ChdC	CPD	HemQ
Coproporphyrinogen decarboxylase	CgdC	PPD	HemF/CPOX
Protoporphyrinogen oxidase	PgoX	PPD	HemY
Protoporphyrin ferrochelatase	PpfC	PPD	HemH
**Heme synthesis from siroheme**			
Uroporphyrinogen methyltransferase	SirA	Siroheme	CysG/CobA
Precorrin-2 dehydrogenase	SirC	Siroheme	CysG
Sirohydrochlorin ferrochelatase	SirB	Siroheme	CysG/CbiX
Siroheme decarboxylase	AhbA	Siroheme	NirD/AhbAB
Siroheme decarboxylase	AhbB	Siroheme	NirH/AhbAB
Didecarboxysiroheme deacetylase	AhbC	Siroheme	NirJ1
Oxygen independent coproheme decarboxylase	AhbD	Siroheme	NirJ2

The biosynthesis of heme via the CPD and PPD can be divided into three parts (1) synthesis of ALA (2) synthesis of coproporphyrinogen III from ALA, and (3) the synthesis of heme from coproporphyrinogen III. The major distinctions in the heme biosynthesis pathway occur in parts 1 and 3, with most bacteria and eukaryotes sharing the pathway from ALA to coproporphyrinogen III (Figure 1).

ALA synthesis is the first committed step for production of bacterial porphyrins found in heme, chlorophyll, cobalamin, and siroheme. Many bacteria synthesize more than one porphyrin derivative, requiring ALA synthesis and subsequent porphyrin synthesis to be regulated to meet multiple metabolic needs. In the Gram-positive bacteria, ALA is synthesized via the C5 pathway which occurs in 2 steps and requires glutamyl-tRNA. Therefore, regulation of ALA synthesis could occur before the heme biosynthesis pathway by regulation of glutamate levels, the loading of glutamyl tRNA via glutamyl tRNA synthetase, or at one of the two pathway enzymes glutamyl tRNA reductase (GtrR) or glutamate-1-semialdehyde aminomutase (GsaM).

From ALA to coproporphyrinogen III the pathways of most bacteria and eukaryotes converge. The penultimate metabolite of this portion of the heme biosynthesis pathway is uroporphyrinogen III. Uroporphyrinogen III is the last intermediate shared between heme synthesis and the synthesis of other porphyrins including chlorophyll, cobalamin, and siroheme (Figure 1). For heme biosynthesis in both the CPD and PPD, uroporphyrinogen III is decarboxylated to yield coproporphyrinogen III, which is the last shared intermediate of these two pathways, and for the final three steps in heme biosynthesis the PPD and CPD diverge.

The PPD is also termed the canonical or classical heme biosynthesis pathway and for many years was considered the only pathway used for heme biosynthesis. In the PPD, coproporphyrinogen III is decarboxylated to yield protoporphyrinogen IX, which is then oxidized to protoporphyrin IX and finally iron is inserted in the last step via protoporphyrin ferrochelatase (PpfC), yielding heme.

In the CPD, coproporphyrinogen III is oxidized to coproporphyrin III, then iron is inserted via coproporphyrin ferrochelatase (CpfC) to yield iron coproheme III, and finally, iron coproheme III is decarboxylated to yield heme. This pathway was designated as noncanonical due to its more recent discovery. The terminal enzyme ChdC in the CPD has no known homologs in the Metazoa, making it of particular interest as a target for possible anti-bacterial therapies (Dailey et al., 2015).

## 2 Regulation of heme synthesis via the coproporphyrin dependent pathway

Herein we discuss the regulation of heme biosynthesis in the Gram-positive phyla, Bacillota (formerly Firmicutes) and Actinomycetota (formerly Actinobacteria). Bacteria of these phyla with known heme biosynthesis regulation mechanisms include the Bacillota *Bacillus subtilis*, *Listeria monocytogenes*, and *Staphylococcus aureus* and the Actinomycetota *Corynebacterium glutamicum* and *Corynebacterium diphtheriae*. The known regulatory mechanisms of these species are varied with heme biosynthesis regulation occurring via iron, oxygen, hydrogen peroxide, and heme, and are summarized in Table 3. The myriad of known regulatory schemes of heme biosynthesis suggests that there may be more regulatory pathways to be discovered in other bacteria from these two phyla, and they preclude the formation of any unifying model for regulation of bacterial heme biosynthesis. Much of the research presented in the literature focuses on regulation of either transcript or protein levels through mechanisms such as transcriptional regulation, post-transcriptional regulation or regulation of protein stability. It is possible that these mechanisms represent only the beginning of our understanding of how bacteria can fine tune heme biosynthesis in response to need. Toward this end, we will also discuss a potential role for protein-protein interactions and PTMs in regulating heme biosynthesis in Gram-positive bacteria.

**TABLE 3:** Summary of heme biosynthesis regulation in the coproporphyrin dependent pathway of the Gram-positive bacteria.

Regulating protein	Species	Enzymes regulated	Regulation	Additional control
**Regulation via heme**				
HemX	*S. aureus* (Choby et al., 2018) *B. subtilis* (Schmitt, 1999)	GtrR	Increased heme levels reduce protein level	
HrrSA	*C. glutamicum* (Frunzke et al., 2011)	GtrR UroD CpfC ChdC	In iron replete conditions heme reduces transcript	DtxR (iron)
HrrSA/ChrSA	*C. diphtheriae* (Bibb et al., 2007; Burgos and Schmitt, 2016)	GtrR	Heme reduces transcript	
**Regulation via iron**				
DtxR	*C. glutamicum* (D’Aquino et al., 2005; Frunzke et al., 2011)	GtrR UroD CpfC ChdC	In iron deplete conditions HrrA represses transcript	HrrSA (heme)
PerR	*B. subtilis* (Fuangthong et al., 2002)	GtrR	Iron levels have slight effect on transcript	PerR (H_2_O_2_)
ideR (putative)	*M. tuberculosis* (Gold et al., 2001; Kurthkoti et al., 2017)	GsaM	Iron levels alter transcript	
unknown	*M. tuberculosis* (Bacon et al., 2007; Kurthkoti et al., 2017)	PbgS CpfC	Iron levels alter transcript	
**Regulation via oxygen and oxidative stress**				
SpxA1	*L. monocytogenes* (Cesinger et al., 2020, 2022)	UroD CpfC	Transcript levels increased in oxidative conditions	
PerR	*B. subtilis* (Chen et al., 1995)	GtrR HemX GsaM PbgS HmbS UroS	Excess H_2_O_2_ increases transcript	
PerR-Iron	*B. subtilis* (Faulkner et al., 2012)	GtrR	H_2_O_2_ increases transcript when PerR is iron bound	
unknown	*M. smegmatis* (Berney and Cook, 2010)	CPD Pathway	Increased transcript in hypoxia along with low O_2_ cytochrome oxidase	
unknown	*M. tuberculosis* (Schubert et al., 2015)	CPD Pathway	Unchanged transcript in hypoxia. Increased transcript of low O_2_ cytochrome oxidase	
unknown	*M. abscessus* (Simcox et al., 2023)	CPD Pathway	Decreased transcript in hypoxia along with decreased transcript of low O_2_ cytochrome oxidase	

### 2.1 Regulation by heme

Feedback inhibition of heme biosynthesis by heme has been reported in the Gram-positive bacteria *B. subtilis*, *S. aureus*, *C. glutamicum*, and *C. diphtheriae*. In the bacteria of the Bacillota phylum, *B. subtilis* and *S. aureus*, heme regulates levels of the first heme synthesis enzyme GtrR via the integral membrane protein HemX (Schröder et al., 1994; Choby et al., 2018). In *S. aureus*, loss of HemX leads to increased levels of synthesized heme and heme precursors (Choby et al., 2018). This unregulated heme synthesis results in the subsequent induction of the heme stress response and disruption of iron homeostasis in these cells, suggesting that HemX is important for regulating heme synthesis in these bacteria in response to heme levels in the cell. The knockout of the hemX gene led to an increase in the level of GtrR protein in these cells and an increase in both intracellular heme levels and in porphyrin intermediates. This buildup of porphyrin and heme would suggest that loss of regulation of GtrR level by HemX is enough to dysregulate porphyrin synthesis and that the late steps of the CPD present a bottleneck to heme biosynthesis in these cells (Choby et al., 2018). The study found that the loss of HemX did not alter transcript level of GtrR, so it is likely that the role of HemX in regulating GtrR protein levels occurs after transcription (Choby et al., 2018). Additionally, while *S. aureus* GtrR can bind heme, the loss of heme binding via GtrR does not alter heme homeostasis in these cells (Leasure et al., 2023) making HemX a necessary component to regulate heme synthesis in response to cellular heme levels in *S. aureus*. Of particular interest in pathogenic bacteria like *S. aureus*, which can utilize exogenous heme in strains lacking proteins of the heme biosynthesis pathway (Skaar, 2010; Mayfield et al., 2013), is how heme uptake and synthesis are regulated. In *S. aureus*, heme uptake is at least in part regulated via iron need by the Fur dependent heme oxygenases (Lojek et al., 2018), however, how *S. aureus* regulates heme synthesis in response to exogenous sources of heme is still not known (Choby et al., 2018). In *B. subtilis*, HemX also regulates the level of GtrR protein in the cells (Schröder et al., 1994). However, in both *S. aureus* and *B. subtilis*, how HemX regulates GtrR protein levels and subsequent heme biosynthesis remains to be discovered.

In the *Corynebacterium*, heme levels regulate heme biosynthesis through the heme-responsive regulator HrrSA two-component system, though the ultimate control of HrrA is under that of the global iron regulator diphtheria toxin repressor (DtxR) (Frunzke et al., 2011). When iron is abundant DtxR represses HrrA and when iron is depleted, HrrA is de-repressed. Studies using a knockout of the response regulator HrrA, found increased transcript levels of four heme biosynthesis enzymes: coproporphyrin ferrochelatase, CpfC (HemH), GtrR (HemA), uroporphyrinogen III decarboxylase, UroD (HemE) and ChdC (HemQ). Which suggests that HrrA represses transcription of these enzymes when iron levels are low. When iron is replete and heme levels are increased, HrrA binds to UroD, however, only CpfC (HemH) transcript level was decreased in wild type cells grown with heme as the iron source (Frunzke et al., 2011). While the exact mechanism of heme dependent HrrA regulation of heme biosynthesis in *C. glutamicum* is not known, in iron deplete cells HrrA suppresses heme biosynthesis to conserve iron, and in iron replete cells HrrA suppresses heme biosynthesis in response to heme levels.

In the pathogen *C. diphtheriae*, heme biosynthesis is regulated through the interplay between the HrrSA and the *Corynebacterium* heme-responsive sensor and activator (ChrSA) two-component systems to repress the transcription of GtrR (hemA) (Bibb et al., 2007). Both HrrSA and ChrSA are activated in response to heme levels (Schmitt, 1999; Burgos and Schmitt, 2016). The systems play a redundant role in regulating GtrR expression as the knockout of HrrSA or ChrSA individually has little effect on GtrR transcription, while loss of both HrrSA and ChrSA leads to an increase in GtrR transcription. In *C. diphtheriae* an exogenous supply of heme as hemoglobin decreases the transcription of GtrR (Bibb et al., 2007; Burgos and Schmitt, 2016). Unlike in the nonpathogenic *C. glutamicum*, GtrR transcript does not appear to be regulated via iron levels (Frunzke et al., 2011). Thus, heme synthesis in these cells is regulated, at least in part, via heme levels through repression of GtrR transcript via the two-component systems HrrSA and ChrSA in *C. diphtheriae*.

In *Mycobacterium tuberculosis*, exogenous heme and Hb can support the growth of cells that do not have functioning heme biosynthesis pathways (Zhang et al., 2020; Donegan et al., 2022). While *M. tuberculosis* has characterized heme uptake pathways (Mitra et al., 2017, 2019; Sankey et al., 2023) and a heme oxygenase enzyme (Nambu et al., 2013; Thakuri et al., 2018; Matthews et al., 2019), the regulation of heme biosynthesis, uptake and degradation is still an active area of investigation. There is some evidence to suggest that heme may regulate heme biosynthesis in mycobacteria. First is that heme binds to the GtrR enzyme (Paravisi et al., 2009) and second exogenous heme reduces the production of intracellular porphyrin intermediates (Donegan et al., 2022). Together this evidence suggests that heme may regulate heme biosynthesis in the mycobacteria, as has been shown for the bacteria discussed above and for others, though more research is needed to understand how this regulation occurs (Dailey et al., 2017; Zamarreño Beas et al., 2022).

### 2.2 Regulation by iron

Given that iron is necessary for heme biosynthesis, iron dependent regulation is a logical point of regulation. In *C. glutamicum*, iron regulates transcript levels of heme biosynthesis proteins indirectly via DtxR (D’Aquino et al., 2005; Frunzke et al., 2011). DtxR is considered to be the main iron-dependent regulator in *C. glutamicum* and regulates the expression of many genes including those associated with iron uptake and storage (D’Aquino et al., 2005; Frunzke et al., 2011). Iron-bound DtxR represses transcript levels of HrrA, which results in the de-repression of GtrR (HemA), UroD (HemE), CpfC (HemH), and ChdC (HemQ) in iron replete conditions in *C. glutamicum* (Wennerhold and Bott, 2006; Frunzke et al., 2011). This interplay between the iron-dependent DtxR and heme-dependent HrrA (see above) suggests a regulatory system that can fine tune heme biosynthesis in these cells to match the levels of heme and iron available both intra- and extracellularly.

In bacteria such as *S. aureus* and *B. subtilis*, a general iron-dependent regulation of heme biosynthesis enzyme levels has not been discovered (Friedman et al., 2006; Choby and Skaar, 2016). Though iron does regulate intracellular heme levels in *S. aureus* by regulating heme uptake and degradation through the iron-regulated surface determinate (Isd) proteins (Skaar and Schneewind, 2004; Choby and Skaar, 2016). In addition, iron binding to a regulatory site on the CpfC enzyme of *S. aureus* and *B. subtilis* reduces enzyme activity and thus downstream heme biosynthesis (Hobbs et al., 2017). In *B. subtilis*, the Fur (ferric uptake repressor) homolog PerR was shown to have a slight effect on GtrR transcript in response to iron, though this is not likely to be the only mechanism of regulation given the low level of response (Fuangthong et al., 2002). So, while iron level has not been shown to have a large effect on enzyme levels in these bacteria, it has a direct effect on enzyme activity of CpfC and at least a small role in regulating heme biosynthesis.

In mycobacteria, some evidence of regulation of heme biosynthesis enzyme transcript level via iron has been shown. In *M. tuberculosis*, the heme biosynthesis enzyme glutamate semialdehyde aminomutase (GsaM), designated as HemL, contains a putative ideR box (Gold et al., 2001) and mRNA levels are decreased in iron limiting media (Kurthkoti et al., 2017). The ferrochelatase CpfC was also found to have reduced mRNA level in iron limiting media (Kurthkoti et al., 2017), however, the porphobilinogen synthase (PbgS), designated as HemB in the paper, was found to have upregulated transcript levels in iron limited media (Bacon et al., 2007). In the mycobacteria, relation of iron level with heme biosynthesis protein level or activity is yet to be determined.

While iron is necessary for the synthesis of heme, the general iron-dependent regulation of heme biosynthesis outside of *C. glutamicum* is not clear. Given that iron must be delivered to CpfC, possibly it is the regulatory cross talk between iron chaperones and heme biosynthesis proteins, like CpfC, that regulates heme biosynthesis. Iron delivery to CpfC could be regulated via a process similar to the interaction between the bacterial frataxin homolog Fra (YdhG) and CpfC as has been discovered in *B. subtilis* (Albrecht et al., 2011) (further discussed below in section entitled “The role of protein-protein interactions in regulation”) and the role of frataxin is well established in eukaryotes (Bencze et al., 2007). Though *in vitro* iron chaperones are not necessary for the iron insertion by PpfC (Yoon and Cowan, 2004) or CpfC (Dali et al., 2023; Gabler et al., 2023), the activity of PpfC is increased in the presence of frataxin (Yoon and Cowan, 2004). Further studies of bacterial frataxin homologs or other potential iron chaperones may provide insight into the regulation of iron insertion via CpfC and subsequent heme biosynthesis in these bacteria.

### 2.3 Regulation via oxygen and oxidative stress

Several of the bacteria that have been discussed are facultative anaerobes, making oxygen availability central to the regulation of heme biosynthesis as heme is needed for aerobic respiration and protection from ROS, and for many Gram-negative bacteria, oxygen availability is a common regulator of heme biosynthesis (Dailey et al., 2017; Zamarreño Beas et al., 2022). Both *L. monocytogenes* and *S. aureus* have been classified as facultative anaerobes, and while *B. subtilis* was originally considered to be strictly aerobic, it has since been discovered to use nitrate as an electron acceptor for growth in anaerobic conditions, making it a likely facultative anaerobe (Hoffmann et al., 1995). In *L. monocytogenes* and *B. subtilis*, heme biosynthesis is regulated in response to oxidative stress. Additionally in *B. subtilis* and *S. aureus*, the synthesis of siroheme from uroporphyrinogen III, which is necessary for nitrate reduction, is regulated via oxygen availability (Nakano et al., 1998; Schlag et al., 2008; Bleul et al., 2022).

*Listeria monocytogenes* regulates the transcript level of two heme biosynthesis enzymes via the SpxA1 transcriptional regulator (Cesinger et al., 2020, 2022). SpxA1 contains a Cysteine-X-X-Cysteine (CXXC) motif which forms an intramolecular disulfide bond under oxidative conditions resulting in the increased transcription of SpxA1 regulated genes (Whiteley et al., 2017). In *L. monocytogenes*, this includes the heme biosynthesis genes uroD (hemE) and cpfC (hemH), which were found to have significantly reduced transcripts (Cesinger et al., 2020) and undetectable protein levels (Cesinger et al., 2022) in an SpxA1 knockout. Conversion of uroporphyrinogen III to coproporphyrinogen III via UroD would dedicate the porphyrin precursors to the synthesis of heme and divert them from the siroheme pathway making UroD a key point of regulation between these two pathways (Figure 1; Dailey et al., 2017). These findings support the upregulation of heme biosynthesis in *L. monocytogenes* in response to oxidative stress.

Generation of hydrogen peroxide occurs during aerobic respiration, and invading pathogens may need to detoxify bursts of hydrogen peroxide from innate immune cells. In *B. subtilis*, mediation of peroxide response and heme biosynthesis is dependent on the peroxide responsive regulator, PerR. PerR is one of three Fur homologs in *B. subtilis* and has been found to be responsive to iron (see above) and manganese in addition to hydrogen peroxide (Fuangthong et al., 2002). Hydrogen peroxide induces heme biosynthesis via upregulation of the operon containing early heme biosynthetic enzymes that synthesize uroporphyrinogen III (hemAXCDBL in text) (Chen et al., 1995). Iron, manganese, and zinc bind PerR, however, only iron-bound PerR regulates GtrR expression in response to hydrogen peroxide levels expected in physiological conditions (Faulkner et al., 2012). An excess of zinc leads to de-repression of heme biosynthesis via PerR and dysregulation of heme biosynthesis to the point of heme toxicity in these cells (Chandrangsu and Helmann, 2016). This is possibly due to the repression of the heme-dependent catalase (KatA), that is both the prominent vegetative catalase in *B. subtilis* and an abundant hemoprotein. KatA is coregulated with heme synthesis in normal zinc levels, but in zinc toxicity, low KatA levels and increased heme biosynthesis possibly lead to heme toxicity (Chandrangsu and Helmann, 2016).

In mycobacteria, the regulation of heme biosynthesis in response to oxygen availability is less well characterized. Mycobacteria are obligate aerobes that can survive and grow during extended periods of hypoxia (Berney and Cook, 2010). In the facultative anaerobes, expression of a heme dependent catalase [e.g., Kat in *L. monocytogenes* (Cesinger et al., 2020), KatA in *B. subtilis* (Fuangthong et al., 2002)] is coregulated with heme biosynthesis in response to oxidative stress. In mycobacteria, KatG is similarly regulated via Fur and induced via hydrogen peroxide (Master et al., 2001). In addition, the expression of KatG in *Mycobacterium bovis* is altered in shaking versus standing conditions, suggesting a link between oxygen levels and regulation (Florczyk et al., 2001). Despite the necessity of heme for an active KatG enzyme, it remains to be seen if the dependence of heme synthesis on oxidative stress is conserved in the obligate aerobes of the mycobacteria. Under low oxygen growth, *Mycobacterium smegmatis* has upregulated heme biosynthesis and upregulated expression of a cytochrome bd oxidase with high affinity for oxygen, allowing the cells to have oxygen dependent respiration even in extremely low oxygen conditions (Berney and Cook, 2010). While this is not under strictly anaerobic conditions, this experiment and the others above suggest that oxygen level may regulate heme biosynthesis to both protect from ROS in regular aerobic growth and to support respiration even in low oxygen in *M. smegmatis*. Though this response may not occur in other mycobacteria. In one study in *M. tuberculosis*, heme biosynthesis enzyme transcript level were mostly unchanged in various hypoxic and reaerated conditions, though the cytochrome bd oxidase was upregulated as was shown in *M. smegmatis* (Schubert et al., 2015). In *M. abscessus*, however, transcript levels of all heme biosynthesis enzymes were decreased in low oxygen levels along with the cytochrome bd oxidase (Simcox et al., 2023). The effect on intracellular heme levels in *M. tuberculosis* and *M. abscessus* during hypoxic growth, however, have yet to be determined. These varied findings suggest that within the mycobacteria, the response to low oxygen levels and subsequent effects on heme biosynthesis vary across species and how this regulation occurs in response to oxygen in the mycobacteria remains to be determined.

## 3 Discussion of future directions

### 3.1 The role of protein-protein interactions in regulation

The enzymes of the CPD pathway are soluble, unlike their membrane bound counterparts in the PPD pathway. This provides strong argument that protein-protein interactions may be necessary to shuttle porphyrin intermediates for heme biosynthesis given their reactivity and potential toxicity (Falb et al., 2023). In addition, the final steps of heme biosynthesis require the delivery of iron to CpfC from iron stores inside the cell or from extracellular sources and the provision of an oxidant, such as hydrogen peroxide produced via CgoX, for the oxidative decarboxylation of iron-coproheme by ChdC to yield heme (Hofbauer et al., 2016; Dailey et al., 2017; Falb et al., 2023).

In mammals, the discovery of the heme metabolon in the mitochondria of erythroid cells provides insight into the potential for protein-protein interactions to support and regulate heme biosynthesis. Here, the terminal heme synthesis enzyme in the PPD, PpfC, interacts with other heme biosynthesis enzymes including the initial pathway enzyme in erythroid cells aminolevulinate synthase 2 (ALAS2) (Medlock et al., 2015; Obi et al., 2022). Many of these proteins, however, are membrane bound and these cells produce higher concentrations of heme than most mammalian cell types, possibly necessitating more permanent interactions between heme biosynthesis proteins. Possibly the interactions of heme biosynthesis proteins in other cells and organisms are more transient. However, as discussed below, there is early evidence for the regulation of heme biosynthesis via protein-protein interactions in Gram-positive bacteria (Table 4).

**TABLE 4:** Protein-protein interactions and post-translational modifications in the coproporphyrin dependent pathway of the Gram-positive bacteria.

Proteins	Species	Interaction measured in native bacteria, *in vitro* or in *E. coli*
**Protein-protein interactions**		
GtrR/Glutamyl-tRNA synthetase (GluRS)	*M. tuberculosis* (Paravisi et al., 2009)	*E. coli*
CpfC/ChdC	*S. aureus* (Celis et al., 2019)	*In vitro* and in *E. coli*
CpfC/ChdC/IsdG (heme oxygenase)	*S. aureus* (Videira et al., 2018)	*E. coli*
Frataxin homolog (Fra)/CpfC	*B. subtilis* (Mielcarek et al., 2015)	In *B. subtilis*
**Enzyme**	**Species**	**Post-translational modifications (PTMs)**
**Post-translational modifications**		
GtrR	*S. aureus*	Phosphorylated (Leasure et al., 2023)
GtrR	*M. smegmatis*	Pupylated (Fascellaro et al., 2016)
PbgS	*S. aureus*	Malonylated (Shi et al., 2021)
UroD	*M. tuberculosis*	Acetylated (Xie et al., 2015) Succinylated (Yang et al., 2015)
CpfC	*S. aureus*	Malonylated (Shi et al., 2021)
ChdC	*S. aureus*	Acetylated (Tan et al., 2022)Succinylated (Tan et al., 2022)
ChdC	*M. tuberculosis*	Pupylated (Festa et al., 2010)

Firstly, the GtrR enzyme of *M. tuberculosis*, designated as GluTR in the paper, has been shown to form a complex with glutamyl–tRNA synthetase (GluRS) when expressed recombinantly in *E. coli* (Paravisi et al., 2009). Intriguingly, all of the soluble GtrR that Paravisi and coworkers were able to isolate was found with bound heme and associated with GluRS (Paravisi et al., 2009). It would be easy to speculate that this interaction could serve to limit initiation of heme biosynthesis when heme is readily available, though whether these interactions occur within *M. tuberculosis* and regulate heme synthesis or if this is the artifact of production in *E. coli* remains to be seen.

In *S. aureus*, the terminal heme biosynthesis enzymes CpfC and ChdC interact both *in vitro* and when expressed recombinantly in *E. coli*, though their role in regulating heme biosynthesis remains to be seen (Videira et al., 2018; Celis and DuBois, 2019; Celis et al., 2019). The interactions between the purified CpfC and ChdC enzymes were found to be transient and did not increase enzyme activity *in vitro* (Celis et al., 2019). In addition, the heme degrading protein IsdG, can bind and reduce CpfC activity (Videira et al., 2018). *S. aureus* expresses two iron-dependent heme oxygenases, IsdG and IsdI that are both regulated in response to iron need via Fur (Reniere and Skaar, 2008). IsdG expression is also regulated via exogenous heme, linking heme biosynthesis directly with acquisition and degradation of heme in these bacteria (Reniere and Skaar, 2008; Videira et al., 2018). Similar to the studies in *M. tuberculosis*, these studies are with either purified enzymes (Celis et al., 2019) or using enzymes expressed in *E. coli* (Videira et al., 2018). Future studies may discover other proteins, including HemX discussed above, that play a role in regulating these interactions in *S. aureus* or in other Gram-positive bacteria.

Finally, initial support for the necessity of protein-protein interactions for the delivery of iron to CpfC has been shown in *B. subtilis* where the bacterial homolog of frataxin (Fra) delivers iron to CpfC. Fra, originally annotated as YdhG in these bacteria, was identified as a frataxin homolog and found to be necessary for supplying iron to the Suf iron sulfur cluster assembly pathway in *B. subtilis* (Albrecht et al., 2011). Later studies revealed an additional role in iron delivery to CpfC for insertion of iron into coproporphyrin III, and an interaction between CpfC and Fra was also identified (Mielcarek et al., 2015). One interesting finding of these studies is that some heme was still produced in cells lacking Fra, revealing the possibility of yet to be discovered auxiliary systems for iron delivery for heme biosynthesis (Mielcarek et al., 2015).

The CPD pathway and the protein-protein interactions involving the heme biosynthesis enzymes of Gram-positive bacteria are relatively recent discoveries. Taken together with the requirement for protection of unstable intermediates and the need for iron delivery, it seems likely that protein-protein interactions will prove to be a key player in the regulation of heme biosynthesis in these bacteria. Future studies that isolate the CPD enzymes and their interacting partners from the native species may prove useful in identifying protein-protein interactions in this pathway as much of the current work looking at potential interactions has been done with purified enzymes. It could be that proteins outside the CPD, post-translational modifications or the presence of metabolites found in the native bacteria are necessary to mediate these interactions.

### 3.2 The role for post-translational modifications in regulation of heme biosynthesis

**FIGURE 2:**
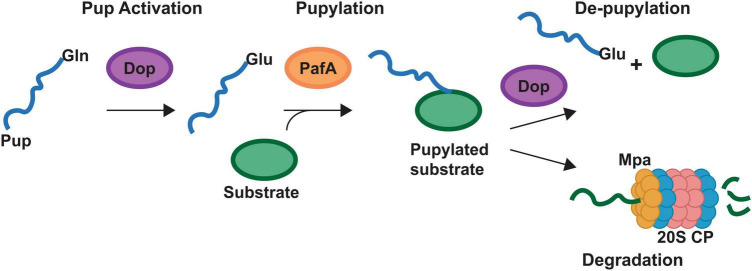
Pupylation pathway of Actinobacteria. Schematic representing the pupylation pathway found in the Actinobacteria. Bacteria may have genes for different prokaryotic ubiquitin protein (Pup) proteins. Designated Pup-Gln (PupQ), Pup-Glu (PupE) or both. PupQ must be deaminated via deaminase of pup (Dop) before being ligated to a substrate protein. Ligation is carried out via the PafA ligase and removal of Pup is carried out via Dop. Mpa recruits pupylated proteins to the 20S proteasome (20S CP) for degradation. Mycobacteria contain the full pathway depicted. Corynebacteria lack the 20S CP.

Improved methodologies for proteomics analysis have aided in the discovery of PTMs of proteins in bacteria. PTMs found on the heme biosynthesis proteins of Gram-positive bacteria include succinylation (Yang et al., 2015; Tan et al., 2022), malonylation (Shi et al., 2021), acetylation (Xie et al., 2015; Tan et al., 2022), pupylation (Pearce et al., 2008; Festa et al., 2010), and phosphorylation (Leasure et al., 2023). These PTMs yield either the addition of an unstructured protein via pupylation, or changes in charge at the protein surface, for succinylation, malonylation, acetylation and phosphorylation. Any of these PTMs may alter protein-ligand or protein-protein interactions. Succinylation, malonylation, or phosphorylation of a lysine would yield a negatively charged modified amino acid residue in place of the positively charged lysine while acetylation would yield a neutral amide. For pupylation, a small ∼ 60 amino acid protein termed prokaryotic ubiquitin-like protein, or Pup, is ligated onto lysine. Similar to the role of ubiquitin in eukaryotes, pupylation can mark proteins for degradation through the Pup proteasome (20S core particle) or can serve to regulate function (Figure 2). While multiple heme biosynthetic enzymes have been found to harbor these PTMs (Table 4), the role of PTMs in regulating heme biosynthesis remains to be discovered. In this section we will discuss the PTMs of the heme biosynthesis proteins of *S. aureus* and various mycobacteria with a perspective on possible implications of these PTMs on heme biosynthesis.

Multiple heme biosynthesis enzymes have been discovered to be malonylated, succinylated, acetylated and phosphorylated (Shi et al., 2021; Tan et al., 2022; Leasure et al., 2023). While the effect of these PTMs on heme biosynthesis is unknown. The alteration of a surface amino acid charge could interrupt or enhance protein-protein or protein-ligand interactions or alter enzyme activity. In *S. aureus*, the CpfC (HemH) enzyme and porphobilinogen synthase, PbgS (HemB) are malonylated (Shi et al., 2021). The terminal enzyme ChdC and IsdG, the heme degradation protein that interacts with CpfC and ChdC (see previous section), are both acetylated and succinylated (Tan et al., 2022). In mycobacteria UroD, which converts uroporphyrinogen III into coproporphyrinogen III is acetylated and succinylated (Xie et al., 2015; Yang et al., 2015). Some mycobacteria can synthesize cobalamin and conversion of uroporphyrinogen III to coproporphyrinogen III would commit the porphyrin ring to the heme biosynthesis pathway, making UroD a potential point of regulation for these two pathways. These modifications at a surface exposed lysine allow for the change in charge of the residue at neutral pH from positive when unmodified, to neutral charge with acetylation or to a negative charge with malonylation or succinylation. In addition, these modifications are reversible and added enzymatically, requiring an acyl CoA-bound metabolite, introducing additional layers of regulation (Yang et al., 2015). In bacteria, these PTMs could offer the ability to quickly fine tune heme biosynthesis in response to changes in metabolic need or environment.

More recently, GtrR from *S. aureus* was discovered to be phosphorylated via the kinase Stk1 (eukaryote-like serine/threonine kinase) and dephosphorylated via the phosphatase Stp1 (eukaryote-like serine/threonine phosphatase) (Leasure et al., 2023). The Stk1/Stp1 pair are involved in the regulation of multiple virulence and growth pathways of *S. aureus* including synthesis of the cell wall, resistance to antibiotics and central metabolism. Loss of Stk1 or Stp1 alone does not directly alter heme levels. However, the knockout of HemX, which regulates GtrR levels in response to heme, in addition to either Stk1 or Stp1 knockout, leads to an increase in heme levels in the cell. Though, heme levels are similar to a ΔhemX background strain, requiring additional studies to link the phosphorylation of GtrR with regulation of heme biosynthesis (Leasure et al., 2023). The metabolic state that determines the phosphorylation of GtrR and the link between heme bound GtrR, HemX and Stk1/Stp1 in regulating heme biosynthesis in *S. aureus* requires further study.

The Actinobacteria possess a PTM not found outside of their phyla termed pupylation which functions similarly to the eukaryotic ubiquitination (Pearce et al., 2008; Striebel et al., 2010, 2014; Barandun et al., 2012). While the protein components are not homologous to the ubiquitin system, the process is similar in that a small protein termed Pup is ligated to proteins at a lysine via a dedicated ligase (PafA, Figure 2). This pupylation can regulate the function of the protein or mark it for degradation via the 20S proteasome (Figure 2). protease (Figure 2). The mycobacteria possess a full Pup proteasomal pathway while the Corynebacteria have the pupylation and de-pupylation enzymes along with the ATPase Mpa but lack the Pup proteasome (Figure 2).

In the mycobacteria, GtrR and ChdC have been found to be pupylated (Festa et al., 2010; Fascellaro et al., 2016). In cells that lack Pup, GtrR levels increase > 2-fold, which suggests that pupylation may regulate GtrR levels and thus porphyrin synthesis (Fascellaro et al., 2016). Interestingly, in the same experiment ChdC levels were unchanged (Fascellaro et al., 2016). This would imply that pupylation of ChdC may be regulatory or the experiment did not use conditions in which ChdC protein level are regulated via Pup. Given that in *S. aureus*, ChdC interacts with CpfC and IsdG, if a similar interaction were necessary in mycobacteria, pupylation at a surface lysine could modulate these interactions. Support for the regulatory role of pupylation comes from the corynebacteria, which have the ability to pupylate and de-pupylate proteins but lack the 20S proteasome (Küberl et al., 2014, 2016). This indicates pupylation in the corynebacteria plays solely a regulatory role. In *C. glutamicum*, pupylation has been shown to regulate iron storage via ferritin (Küberl et al., 2014, 2016). The release of iron via ferritin occurs when individual ferritin monomers are pupylated and depupylation via deaminase of Pup (Dop, Figure 2) can allow the ferritin monomers to reassemble and store iron.

**FIGURE 3:**
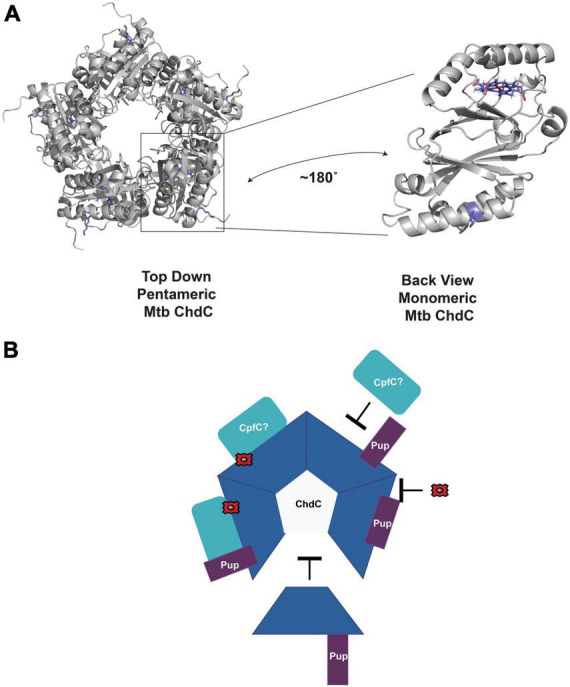
Potential role of pupylation in regulating enzyme activity. **(A)** AlphaFold model of *M. tuberculosis* ChdC monomer aligned as a pentamer using PDB ID 5LOQ. Coproheme is also modeled from alignment with PDB ID 5LOQ. The pupylated Lys44 is in blue sticks. The coproheme modeled in the active site is also in blue sticks. A monomer is rotated to highlight the location of Lys44 and the active site of ChdC. **(B)** Some of the possible roles of Pup in regulating mycobacterial ChdC activity. Pup may inhibit or enhance interactions with CpfC or other proteins. Pup may inhibit oligomerization to pentameric ChdC. Pup may alter protein ligand interactions such as with iron-coproheme. All of these are unproven hypothesis of the role in Pup dependent ChdC regulation.

Using the model of *M. tuberculosis* monomer ChdC generated from AlphaFold (Jumper et al., 2021; Varadi et al., 2022), aligned to the pentameric *Listeria monocytogenes* structure from the PDB (Hofbauer et al., 2016) (PDB ID 5LOQ), we see that the Lys44 pupylated in ChdC is at the surface as expected (Figure 3A; Festa et al., 2010). The location of Lys44 in the model is on the same side as the active site for heme synthesis which is modeled with the coproheme from the 5LOQ structure in Figure 3. This brings to mind many possibilities for how pupylation could modulate ChdC activity. The pup protein could act as a gate to regulate substrate binding or product release, alter oligomerization as with the *C. glutamicum* ferritin, or modulate protein-protein interactions among other possibilities (Figure 3B).

The relatively recent discoveries of the CPD along with the PTMs of heme biosynthesis enzymes has opened a vast array of possibilities for heme biosynthesis regulation. However, much more research is needed to understand the roles, if any, for succinylation, malonylation, acetylation, phosphorylation and pupylation in regulating heme biosynthesis in the Gram-positive bacteria. Each new discovery, however, has provided testable hypotheses for the role of these PTMs, and future research will clarify how heme biosynthesis is regulated in these bacteria.

### 3.3 Targeting the coproporphyrin dependent pathway

Until the discovery of ChdC and the CPD pathway for heme biosynthesis, it was generally considered that heme biosynthesis in humans and bacteria shared a common pathway from ALA. Therefore, any drug that targeted heme biosynthesis in pathogenic bacteria was likely to interfere with heme biosynthesis in the human host as well. With the discovery of ChdC, which has no known human homolog, the possibility of targeting heme biosynthesis in pathogens of the Gram-positive bacteria became more feasible (Dailey et al., 2015). Recently, a drug screen comparing the growth of two strains of *E. coli*, a native strain that uses the PPD, and a strain with the PPD replaced with the enzymes of the *S. aureus* CPD was used to screen a drug library to identify compounds that specifically targeted heme biosynthesis via the CPD (Jackson et al., 2023). Of the five identified compounds from the screen, three were active against *S. aureus*, likely by inhibition of ChdC. This screen validates ChdC as a potential anti-bacterial target and opens up the possibility of targeting heme biosynthesis in bacteria the utilize the CPD pathway.

## 4 Conclusion

There does not appear to be one uniting regulatory mechanism for heme biosynthesis in the Gram-positive bacteria. This is not surprising given the necessity of heme in different environments, the shared pathway from ALA to uroporphyrinogen with other metabolites, and the requirement of glutamyl tRNA and iron for synthesis. With so many pathways entwined with heme biosynthesis, there are likely many junctures at which heme biosynthesis can be regulated. Some pressing questions have emerged from the discovery of some regulatory mechanisms. For example, how and under what conditions is porphyrin diverted from heme synthesis to vitamin B12 synthesis in organisms that make both? How is iron delivered to CpfC and are there different routes for imported iron versus iron from storage proteins like ferritin? What is the role of PTMs of heme biosynthesis enzymes in regulating heme biosynthesis? Does heme biosynthesis via soluble cytosolic proteins require some method of substate shuttling via either protein-protein interactions or unidentified chaperones? Most importantly, as we learn more about the regulation of heme biosynthesis in these bacteria, we may identify new drug targets to combat the multitude of pathogens within these phyla.

## Author contributions

HA: Writing – original draft, Writing – review & editing. RD: Writing – original draft, Writing – review & editing.

## References

[B1] Albrecht A. G. Landmann H. Nette D. Burghaus O. Peuckert F. Seubert A. (2011). The frataxin homologue fra plays a key role in intracellular iron channeling in Bacillus subtilis. *ChemBioChem* 12 2052–2061. 10.1002/cbic.201100190 21744456

[B2] Bacon J. Dover L. G. Hatch K. A. Zhang Y. Gomes J. M. Kendall S. (2007). Lipid composition and transcriptional response of Mycobacterium tuberculosis grown under iron-limitation in continuous culture: identification of a novel wax ester. *Microbiology* 153(Pt 5), 1435–1444. 10.1099/mic.0.2006/004317-0 17464057 PMC3123377

[B3] Barandun J. Delley C. L. Weber-Ban E. (2012). The pupylation pathway and its role in mycobacteria. *BMC Biol.* 10:95. 10.1186/1741-7007-10-95 23198822 PMC3511204

[B4] Bencze K. Z. Yoon T. Millán-Pacheco C. Bradley P. B. Pastor N. Cowan J. A. (2007). Human frataxin: iron and ferrochelatase binding surface. *Chem. Commun.* 18 1798–1800. 10.1039/b703195e PMC2862461 17476391

[B5] Berney M. Cook G. M. (2010). Unique flexibility in energy metabolism allows mycobacteria to combat starvation and hypoxia. *PLoS One* 5:e8614. 10.1371/journal.pone.0008614 20062806 PMC2799521

[B6] Bibb L. A. Kunkle C. A. Schmitt M. P. (2007). The ChrA-ChrS and HrrA-HrrS signal transduction systems are required for activation of the hmuO promoter and repression of the hemA promoter in Corynebacterium diphtheriae. *Infect. Immun.* 75 2421–2431. 10.1128/IAI.01821-06 17353293 PMC1865786

[B7] Bleul L. Francois P. Wolz C. (2022). Two-component systems of S. aureus: signaling and sensing mechanisms. *Genes* 13:34. 10.3390/genes13010034 PMC8774646 35052374

[B8] Burgos J. M. Schmitt M. P. (2016). The ChrSA and HrrSA two-component systems are required for transcriptional regulation of the hema promoter in Corynebacterium diphtheriae. *J. Bacteriol.* 198 2419–2430. 10.1128/JB.00339-16 27381918 PMC4999929

[B9] Celis A. I. Choby J. E. Kentro J. Skaar E. P. DuBois J. L. (2019). Control of metabolite flux during the final steps of heme b biosynthesis in Gram positive bacteria. *Biochemistry* 58 5259–5270. 10.1021/acs.biochem.9b00140 31241911 PMC7160669

[B10] Celis A. I. DuBois J. L. (2019). Making and breaking heme. *Curr. Opin. Struct. Biol.* 59 19–28. 30802830 10.1016/j.sbi.2019.01.006 PMC6706330

[B11] Cesinger M. R. Daramola O. I. Kwiatkowski L. M. Reniere M. L. (2022). The transcriptional regulator SpxA1 influences the morphology and virulence of listeria monocytogenes. *Infect. Immun.* 90:e00211-22. 10.1128/iai.00211-22 36102657 PMC9584327

[B12] Cesinger M. R. Thomason M. K. Edrozo M. B. Halsey C. R. Reniere M. L. (2020). Listeria monocytogenes SpxA1 is a global regulator required to activate genes encoding catalase and heme biosynthesis enzymes for aerobic growth. *Mol. Microbiol.* 114 230–243. 10.1111/mmi.14508 32255216 PMC7496741

[B13] Chandrangsu P. Helmann J. D. (2016). Intracellular Zn(II) intoxication leads to dysregulation of the perr regulon resulting in heme toxicity in Bacillus subtilis. *PLoS Genet.* 12:e1006515. 10.1371/journal.pgen.1006515 27935957 PMC5189952

[B14] Chen L. Keramati L. Helmann J. D. (1995). Coordinate regulation of Bacillus subtilis peroxide stress genes by hydrogen peroxide and metal ions. *Proc. Natl. Acad. Sci.* 92 8190–8194. 7667267 10.1073/pnas.92.18.8190 PMC41122

[B15] Choby J. E. Grunenwald C. M. Celis A. I. Gerdes S. Y. DuBois J. L. Skaar E. P. (2018). Staphylococcus aureus HemX modulates glutamyl-tRNA reductase abundance to regulate heme biosynthesis. *MBio* 9:e02287-17. 10.1128/mBio.02287-17 29437922 PMC5801465

[B16] Choby J. E. Skaar E. P. (2016). Heme synthesis and acquisition in bacterial pathogens. *J. Mol. Biol.* 428 3408–3428. 27019298 10.1016/j.jmb.2016.03.018 PMC5125930

[B17] Dailey H. A. Dailey T. A. Gerdes S. Jahn D. Jahn M. O’Brian M. R. (2017). Prokaryotic heme biosynthesis: multiple pathways to a common essential product. *Microbiol. Mol. Biol. Rev.* 81:e00048-16. 10.1128/MMBR.00048-16 28123057 PMC5312243

[B18] Dailey H. A. Gerdes S. Dailey T. A. Burch J. S. Phillips J. D. (2015). Noncanonical coproporphyrin-dependent bacterial heme biosynthesis pathway that does not use protoporphyrin. *Proc. Natl. Acad. Sci.* 112 2210–2215. 10.1073/pnas.1416285112 25646457 PMC4343137

[B19] Dailey H. A. Medlock A. E. (2022). A primer on heme biosynthesis. *Biol. Chem.* 403 985–1003. 36029525 10.1515/hsz-2022-0205

[B20] Dailey T. A. Boynton T. O. Albetel A.-N. Gerdes S. Johnson M. K. Dailey H. A. (2010). Discovery and characterization of HemQ an essential heme biosynthetic pathway component. *J. Biol. Chem.* 285 25978–25986. 10.1074/jbc.M110.142604 20543190 PMC2923992

[B21] Dali A. Gabler T. Sebastiani F. Destinger A. Furtmüller P. G. Pfanzagl V. (2023). Active site architecture of coproporphyrin ferrochelatase with its physiological substrate coproporphyrin III: propionate interactions and porphyrin core deformation. *Protein Sci.* 32:e4534. 10.1002/pro.4534 36479958 PMC9794026

[B22] D’Aquino J. A. Tetenbaum-Novatt J. White A. Berkovitch F. Ringe D. (2005). Mechanism of metal ion activation of the diphtheria toxin repressor DtxR. *Proc. Natl. Acad. Sci.* 102 18408–18413. 16352732 10.1073/pnas.0500908102 PMC1317899

[B23] Donegan R. K. Fu Y. Copeland J. Idga S. Brown G. Hale O. F. (2022). Exogenously scavenged and endogenously synthesized heme are differentially utilized by Mycobacterium tuberculosis. *Microbiol. Spectrum* 10:e0360422. 10.1128/spectrum.03604-22 36169423 PMC9604157

[B24] Donegan R. K. Moore C. M. Hanna D. A. Reddi A. R. (2019). Handling heme: the mechanisms underlying the movement of heme within and between cells. *Free Radical Biol. Med.* 133 88–100. 30092350 10.1016/j.freeradbiomed.2018.08.005 PMC6363905

[B25] Falb N. Patil G. Furtmüller P. G. Gabler T. Hofbauer S. (2023). Structural aspects of enzymes involved in prokaryotic gram-positive heme biosynthesis. *Comput. Struct. Biotechnol. J.* 21 3933–3945. 10.1016/j.csbj.2023.07.024 37593721 PMC10427985

[B26] Fascellaro G. Petrera A. Lai Z. W. Nanni P. Grossmann J. Burger S. (2016). Comprehensive proteomic analysis of nitrogen-starved Mycobacterium smegmatis Δ pup reveals the impact of pupylation on nitrogen stress response. *J. Proteome Res.* 15 2812–2825. 10.1021/acs.jproteome.6b00378 27378031

[B27] Faulkner M. J. Ma Z. Fuangthong M. Helmann J. D. (2012). Derepression of the Bacillus subtilis PerR peroxide stress response leads to iron deficiency. *J. Bacteriol.* 194 1226–1235. 10.1128/JB.06566-11 22194458 PMC3294777

[B28] Festa R. A. McAllister F. Pearce M. J. Mintseris J. Burns K. E. Gygi S. P. (2010). Prokaryotic ubiquitin-like protein (Pup) proteome of Mycobacterium tuberculosis [corrected]. *PLoS One* 5:e8589. 10.1371/journal.pone.0008589 20066036 PMC2797603

[B29] Florczyk M. A. McCue L. A. Stack R. F. Hauer C. R. McDonough K. A. (2001). Identification and characterization of mycobacterial proteins differentially expressed under standing and shaking culture conditions, including Rv2623 from a novel class of putative ATP-binding proteins. *Infect. Immun.* 69 5777–5785. 10.1128/IAI.69.9.5777-5785.2001 11500455 PMC98695

[B30] Friedman D. B. Stauff D. L. Pishchany G. Whitwell C. W. Torres V. J. Skaar E. P. (2006). Staphylococcus aureus redirects central metabolism to increase iron availability. *PLoS Pathog.* 2:e87. 10.1371/journal.ppat.0020087 16933993 PMC1557832

[B31] Frunzke J. Gätgens C. Brocker M. Bott M. (2011). Control of heme homeostasis in *Corynebacterium glutamicum* by the two-component system HrrSA. *J. Bacteriol.* 193 1212–1221. 10.1128/JB.01130-10 21217007 PMC3067591

[B32] Fuangthong M. Herbig A. F. Bsat N. Helmann J. D. (2002). Regulation of the Bacillus subtilis fur and perR genes by PerR: not all members of the PerR regulon are peroxide inducible. *J. Bacteriol.* 184 3276–3286. 12029044 10.1128/JB.184.12.3276-3286.2002 PMC135084

[B33] Gabler T. Dali A. Sebastiani F. Furtmüller P. G. Becucci M. Hofbauer S. (2023). Iron insertion into coproporphyrin III-ferrochelatase complex: evidence for an intermediate distorted catalytic species. *Protein Sci.* 32:e4788. 10.1002/pro.4788 37743577 PMC10578119

[B34] Gold B. Rodriguez G. M. Marras S. A. E. Pentecost M. Smith I. (2001). The Mycobacterium tuberculosis IdeR is a dual functional regulator that controls transcription of genes involved in iron acquisition, iron storage and survival in macrophages. *Mol. Microbiol.* 42 851–865. 10.1046/j.1365-2958.2001.02684.x 11722747

[B35] Hobbs C. Reid J. D. Shepherd M. (2017). The coproporphyrin ferrochelatase of Staphylococcus aureus: mechanistic insights into a regulatory iron-binding site. *Biochem. J.* 474 3513–3522. 10.1042/BCJ20170362 28864672 PMC5633918

[B36] Hofbauer S. Mlynek G. Milazzo L. Pühringer D. Maresch D. Schaffner I. (2016). Hydrogen peroxide-mediated conversion of coproheme to heme b by HemQ—lessons from the first crystal structure and kinetic studies. *FEBS J.* 283 4386–4401. 10.1111/febs.13930 27758026 PMC5157759

[B37] Hoffmann T. Troup B. Szabo A. Hungerer C. Jahn D. (1995). The anaerobic life of Bacillus subtilis: cloning of the genes encoding the respiratory nitrate reductase system. *FEMS Microbiol. Lett.* 131 219–225. 10.1111/j.1574-6968.1995.tb07780.x 7557333

[B38] Jackson L. K. Dailey T. A. Anderle B. Warren M. J. Bergonia H. A. Dailey H. A. (2023). Exploiting differences in heme biosynthesis between bacterial species to screen for novel antimicrobials. *Biomolecules* 13:1485. 10.3390/biom13101485 37892169 PMC10604556

[B39] Jumper J. Evans R. Pritzel A. Green T. Figurnov M. Ronneberger O. (2021). Highly accurate protein structure prediction with AlphaFold. *Nature* 596 583–589. 34265844 10.1038/s41586-021-03819-2 PMC8371605

[B40] Kim S. Kang I. Lee J.-W. Jeon C. O. Giovannoni S. J. Cho J.-C. (2021). Heme auxotrophy in abundant aquatic microbial lineages. *Proc. Natl. Acad. Sci.* 118:e2102750118. 10.1073/pnas.2102750118 34785591 PMC8617464

[B41] Küberl A. Fränzel B. Eggeling L. Polen T. Wolters D. A. Bott M. (2014). Pupylated proteins in Corynebacterium glutamicum revealed by MudPIT analysis. *Proteomics* 14 1531–1542. 10.1002/pmic.201300531 24737727

[B42] Küberl A. Polen T. Bott M. (2016). The pupylation machinery is involved in iron homeostasis by targeting the iron storage protein ferritin. *Proc. Natl. Acad. Sci.* 113 4806–4811. 10.1073/pnas.1514529113 27078093 PMC4855571

[B43] Kurthkoti K. Amin H. Marakalala M. J. Ghanny S. Subbian S. Sakatos A. (2017). The capacity of mycobacterium tuberculosis to survive iron starvation might enable it to persist in iron-deprived microenvironments of human granulomas. *mBio* 8:e01092-17. 10.1128/mBio.01092-17 28811344 PMC5559634

[B44] Layer G. (2021). Heme biosynthesis in prokaryotes. *Biochim. Biophys. Acta Mol. Cell Res.* 1868:118861. 10.1016/j.bbamcr.2020.118861 32976912

[B45] Leasure C. S. Grunenwald C. M. Choby J. E. Sauer J.-D. Skaar E. P. (2023). Maintenance of heme homeostasis in Staphylococcus aureus through post-translational regulation of glutamyl-tRNA reductase. *J. Bacteriol.* 205:e00171123. 10.1128/jb.00171-23 37655914 PMC10521356

[B46] Lojek L. J. Farrand A. J. Weiss A. Skaar E. P. (2018). Fur regulation of Staphylococcus aureus heme oxygenases is required for heme homeostasis. *Int. J. Med. Microbiol.* 308 582–589. 10.1016/j.ijmm.2018.01.009 29409696 PMC6070430

[B47] Master S. Zahrt T. C. Song J. Deretic V. (2001). Mapping of Mycobacterium tuberculosis katG promoters and their differential expression in infected macrophages. *J. Bacteriol.* 183 4033–4039. 10.1128/JB.183.13.4033-4039.2001 11395468 PMC95287

[B48] Matthews S. J. Pacholarz K. J. France A. P. Jowitt T. A. Hay S. Barran P. E. (2019). MhuD from Mycobacterium tuberculosis: probing a dual role in heme storage and degradation. *ACS Infectious Dis.* 5 1855–1866. 10.1021/acsinfecdis.9b00181 31480841

[B49] Mayfield J. A. Hammer N. D. Kurker R. C. Chen T. K. Ojha S. Skaar E. P. (2013). The chlorite dismutase (HemQ) from Staphylococcus aureus has a redox-sensitive heme and is associated with the small colony variant phenotype. *J. Biol. Chem.* 288 23488–23504. 10.1074/jbc.M112.442335 23737523 PMC5395028

[B50] Medlock A. E. Shiferaw M. T. Marcero J. R. Vashisht A. A. Wohlschlegel J. A. Phillips J. D. (2015). Identification of the mitochondrial heme metabolism complex. *PLoS One* 10:e0135896. 10.1371/journal.pone.0135896 26287972 PMC4545792

[B51] Mielcarek A. Blauenburg B. Miethke M. Marahiel M. A. (2015). Molecular insights into frataxin-mediated iron supply for heme biosynthesis in Bacillus subtilis. *PLoS One* 10:e0122538. 10.1371/journal.pone.0122538 25826316 PMC4380498

[B52] Mitra A. Ko Y.-H. Cingolani G. Niederweis M. (2019). Heme and hemoglobin utilization by Mycobacterium tuberculosis. *Nat. Commun.* 10:4260. 10.1038/s41467-019-12109-5 PMC6751184 31534126

[B53] Mitra A. Speer A. Lin K. Ehrt S. Niederweis M. (2017). PPE surface proteins are required for heme utilization by Mycobacterium tuberculosis. *mBio* 8:e01720-16. 10.1128/mBio.01720-16 PMC5263243 28119467

[B54] Nakano M. M. Hoffmann T. Zhu Y. Jahn D. (1998). Nitrogen and oxygen regulation of Bacillus subtilis nasDEF encoding NADH-Dependent nitrite reductase by TnrA and ResDE. *J. Bacteriol.* 180 5344–5350. 10.1128/JB.180.20.5344-5350.1998 9765565 PMC107582

[B55] Nambu S. Matsui T. Goulding C. W. Takahashi S. Ikeda-Saito M. (2013). A new way to degrade heme the Mycobacterium tuberculosis enzyme MhuD catalyzes heme degradation without generating CO. *J. Biol. Chem.* 288 10101–10109. 10.1074/jbc.M112.448399 23420845 PMC3617252

[B56] Obi C. D. Bhuiyan T. Dailey H. A. Medlock A. E. (2022). Ferrochelatase: mapping the intersection of iron and porphyrin metabolism in the mitochondria. *Front. Cell Dev. Biol.* 10:894591. 10.3389/fcell.2022.894591 35646904 PMC9133952

[B57] Paravisi S. Fumagalli G. Riva M. Morandi P. Morosi R. Konarev P. V. (2009). Kinetic and mechanistic characterization of Mycobacterium tuberculosis glutamyl–tRNA synthetase and determination of its oligomeric structure in solution. *FEBS J.* 276 1398–1417. 10.1111/j.1742-4658.2009.06880.x 19187240

[B58] Pearce M. J. Mintseris J. Ferreyra J. Gygi S. P. Darwin K. H. (2008). Ubiquitin-Like protein involved in the proteasome pathway of Mycobacterium tuberculosis. *Science* 322 1104–1107. 18832610 10.1126/science.1163885 PMC2698935

[B59] Reniere M. L. Skaar E. P. (2008). Staphylococcus aureus haem oxygenases are differentially regulated by iron and haem. *Mol. Microbiol.* 69 1304–1315. 18643935 10.1111/j.1365-2958.2008.06363.x PMC2597461

[B60] Sankey N. Merrick H. Singh P. Rogers J. Reddi A. Hartson S. D. (2023). Role of the Mycobacterium tuberculosis ESX-4 secretion system in heme iron utilization and pore formation by PPE proteins. *mSphere* 8:e0057322. 10.1128/msphere.00573-22 36749044 PMC10117145

[B61] Schlag S. Fuchs S. Nerz C. Gaupp R. Engelmann S. Liebeke M. (2008). Characterization of the oxygen-responsive NreABC regulon of Staphylococcus aureus. *J. Bacteriol.* 190 7847–7858. 10.1128/JB.00905-08 18820014 PMC2583599

[B62] Schmitt M. P. (1999). Identification of a two-component signal transduction system from corynebacterium diphtheriae that activates gene expression in response to the presence of heme and hemoglobin. *J. Bacteriol.* 181 5330–5340. 10.1128/JB.181.17.5330-5340.1999 10464204 PMC94039

[B63] Schröder I. Johansson P. Rutberg L. Hederstedt L. (1994). The hemX gene of the Bacillus subtilis hemAXCDBL operon encodes a membrane protein, negatively affecting the steady-state cellular concentration of HemA (glutamyl-tRNA reductase). *Microbiology* 140 731–740. 10.1099/00221287-140-4-731 8012594

[B64] Schubert O. T. Ludwig C. Kogadeeva M. Zimmermann M. Rosenberger G. (2015). Absolute proteome composition and dynamics during dormancy and resuscitation of Mycobacterium tuberculosis. *Cell Host Microbe* 18 96–108. 10.1016/j.chom.2015.06.001 26094805

[B65] Shi Y. Zhu J. Xu Y. Tang X. Yang Z. Huang A. (2021). Malonyl-proteome profiles of Staphylococcus aureus reveal lysine malonylation modification in enzymes involved in energy metabolism. *Proteome Sci.* 19:1. 10.1186/s12953-020-00169-1 33436009 PMC7802289

[B66] Simcox B. S. Tomlinson B. R. Shaw L. N. Rohde K. H. (2023). Mycobacterium abscessus DosRS two-component system controls a species-specific regulon required for adaptation to hypoxia. *Front. Cell. Infect. Microbiol.* 13:1144210. 10.3389/fcimb.2023.1144210 36968107 PMC10034137

[B67] Skaar E. P. (2010). The battle for iron between bacterial pathogens and their vertebrate hosts. *PLoS Pathog.* 6:e1000949. 10.1371/journal.ppat.1000949 20711357 PMC2920840

[B68] Skaar E. P. Schneewind O. (2004). Iron-regulated surface determinants (Isd) of Staphylococcus aureus: stealing iron from heme. *Microbes Infect*. 6 390–397. 15101396 10.1016/j.micinf.2003.12.008

[B69] Striebel F. Hunkeler M. Summer H. Weber-Ban E. (2010). The mycobacterial Mpa-proteasome unfolds and degrades pupylated substrates by engaging Pup’s N-terminus. *EMBO J.* 29 1262–1271. 10.1038/emboj.2010.23 20203624 PMC2857465

[B70] Striebel F. Imkamp F. Özcelik D. Weber-Ban E. (2014). Pupylation as a signal for proteasomal degradation in bacteria. *Biochim. Biophys. Acta Mol. Cell Res.* 1843 103–113. 10.1016/j.bbamcr.2013.03.022 23557784

[B71] Tan L. Yang Y. Shang W. Hu Z. Peng H. Li S. (2022). Identification of Lysine Succinylome and acetylome in the vancomycin-intermediate Staphylococcus aureus XN108. *Microbiol. Spectr.* 10:e0348122. 10.1128/spectrum.03481-22 36374118 PMC9769639

[B72] Thakuri B. Graves A. B. Chao A. Johansen S. L. Goulding C. W. Liptak M. D. (2018). The affinity of MhuD for heme is consistent with a heme degrading function in vivo. *Metallomics* 10 1560–1563. 10.1039/c8mt00238j 30239544 PMC6529808

[B73] Varadi M. Anyango S. Deshpande M. Nair S. Natassia C. Yordanova G. (2022). AlphaFold protein structure database: massively expanding the structural coverage of protein-sequence space with high-accuracy models. *Nucleic Acids Res.* 50 D439–D444. 10.1093/nar/gkab1061 34791371 PMC8728224

[B74] Videira M. A. Lobo S. A. Silva L. S. Palmer D. J. Warren M. J. Prieto M. (2018). Staphylococcus aureus haem biosynthesis and acquisition pathways are linked through haem monooxygenase IsdG. *Mol. Microbiol.* 109 385–400. 10.1111/mmi.14060 29989674

[B75] Wennerhold J. Bott M. (2006). The DtxR regulon of Corynebacterium glutamicum. *J. Bacteriol.* 188 2907–2918. 16585752 10.1128/JB.188.8.2907-2918.2006 PMC1446976

[B76] Whiteley A. T. Ruhland B. R. Edrozo M. B. Reniere M. L. (2017). A redox-responsive transcription factor is critical for pathogenesis and aerobic growth of Listeria monocytogenes. *Infect. Immun.* 85:e00978-16. 10.1128/IAI.00978-16 28193635 PMC5400837

[B77] Xie L. Wang X. Zeng J. Zhou M. Duan X. Li Q. (2015). Proteome-wide lysine acetylation profiling of the human pathogen Mycobacterium tuberculosis. *Int. J. Biochem. Cell Biol.* 59 193–202. 10.1016/j.biocel.2014.11.010 25456444

[B78] Yang M. Wang Y. Chen Y. Cheng Z. Gu J. Deng J. (2015). Succinylome analysis reveals the involvement of lysine succinylation in metabolism in pathogenic Mycobacterium tuberculosis. *Mol. Cell Proteomics* 14 796–811. 10.1074/mcp.M114.045922 25605462 PMC4390261

[B79] Yoon T. Cowan J. A. (2004). Frataxin-mediated iron delivery to ferrochelatase in the final step of heme biosynthesis. *J. Biol. Chem.* 279 25943–25946. 10.1074/jbc.C400107200 15123683

[B80] Zamarreño Beas J. Videira M. A. M. Saraiva L. M. (2022). Regulation of bacterial haem biosynthesis. *Coordination Chem. Rev.* 452:214286.

[B81] Zámocký M. Hofbauer S. Gabler T. Furtmüller P. G. (2023). The molecular evolution, structure, and function of coproporphyrinogen oxidase and protoporphyrinogen oxidase in prokaryotes. *Biology* 12:1527. 10.3390/biology12121527 38132353 PMC10740692

[B82] Zhang L. Hendrickson R. C. Meikle V. Lefkowitz E. J. Ioerger T. R. Niederweis M. (2020). Comprehensive analysis of iron utilization by Mycobacterium tuberculosis. *PLoS Pathog.* 16:e1008337. 10.1371/journal.ppat.1008337 32069330 PMC7058343

